# Evaluate the efficacy and reliability of functional gradients in within‐subject designs

**DOI:** 10.1002/hbm.26213

**Published:** 2023-01-20

**Authors:** Xiaolong Zhang, Zhenxiang Zang

**Affiliations:** ^1^ Department of Physiology, College of Basic Medical Sciences Army Medical University Chongqing China; ^2^ Department of Radiology and Nuclear Medicine, Xuanwu Hospital Capital Medical University Beijing China; ^3^ Beijing Key Laboratory of Magnetic Resonance Imaging and Brain Informatics Beijing China

**Keywords:** eyes open and eyes closed, gradients, reliability, within‐subject

## Abstract

The cerebral cortex is characterized as the integration of distinct functional principles that correspond to basic primary functions, such as vision and movement, and domain‐general functions, such as attention and cognition. Diffusion embedding approach is a novel tool to describe transitions between different functional principles, and has been successively applied to investigate pathological conditions in between‐group designs. What still lacking and urgently needed is the efficacy of this method to differentiate within‐subject circumstances. In this study, we applied the diffusion embedding to eyes closed (EC) and eyes on (EO) resting‐state conditions from 145 participants. We found significantly lower within‐network dispersion of visual network (VN) (*p* = 7.3 × 10^−4^) as well as sensorimotor network (SMN) (*p* = 1 × 10^−5^) and between‐network dispersion of VN (*p* = 2.3 × 10^−4^) under EC than EO, while frontoparietal network (*p* = 9.2 × 10^−4^) showed significantly higher between‐network dispersion during EC than EO. Test–retest reliability analysis further displayed fair reliability (intraclass correlation coefficient [ICC] < 0.4) of the network dispersions (mean ICC = 0.116 ± 0.143 [standard deviation]) except for the within‐network dispersion of SMN under EO (ICC = 0.407). And the reliability under EO was higher but not significantly higher than reliability under EC. Our study demonstrated that the diffusion embedding approach that shows fair reliability is capable of distinguishing EC and EO resting‐state conditions, such that this method could be generalized to other within‐subject designs.

## Graphical abstract

Diffusion embedding can differentiate within‐subject conditions‐eyes closed and open. Visual, sensorimotor and frontoparietal network showed significant differences. Network dispersion showed fair reliability.

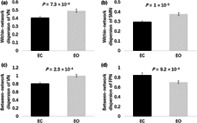

## 1 INTRODUCTION

The topology of the cerebral cortex offers an organization principle that constrains its cognitive processes, which is a key assumption in neuroscience. Recent advances in the domain of human connectome have identified a number of large‐scale networks of the cerebral cortex (Damoiseaux et al., 2006; Power et al., 2011; Yeo et al., 2011), such as visual network (VN) and salience network, each characterized by distinct functional profiles. Some are associated with basic primary functions, such as vision (Katzner & Weigelt, 2013) and movement (Svoboda & Li, 2018); some serve domain‐general functions, such as cognitive control (Bressler & Menon, 2010) and attention (Fiebelkorn & Kastner, 2020).

Diffusion embedding is a novel tool to investigate the functionally topographical structure and the organizing spatial framework of the large‐scale brain networks. Through projecting high‐dimension connectomes into lower‐dimension manifold spaces, it can visualize biologically meaningful principal gradients of cortical network organization. These gradients depict a functional hierarchy of cortical organization with primary motor/sensory regions at one end and transmodal regions such as default mode network (DMN) at the other end (Margulies et al., 2016). This method has been widely used in between‐subject conditions to explore the functional gradients of cortex (Paquola et al., 2019) as well as cerebellum (Guell et al., 2018), the development of functional brain architecture (Bethlehem et al., 2020; Dong et al., 2021), and atypical functional organization in brain diseases (Caciagli et al., 2022; Hong et al., 2019). But whether it could efficiently differentiate the within‐subject conditions is unclear.

Functional gradients depend on decomposing the high‐dimension connectivity matrix (Margulies et al., 2016) and thus are suitable for resting‐state data (Bethlehem et al., 2020; Dong et al., 2021; Hong et al., 2019). A critical reason is that resting state engages relatively consistent brain spontaneous activity patterns, making it methodologically suitable for the construction of a connectivity matrix (Chen et al., 2020). In addition, eyes open (EO) and eyes closed (EC) are two common resting‐state conditions that display robust differences in brain activity as well as connectivity patterns within several large‐scale brain networks. For example, previous studies have investigated the differences using methods including activation (Ben‐Simon et al., 2008), regional homogeneity (Wei et al., 2018), amplitude of low‐frequency fluctuation (Yan et al., 2009; Zhang et al., 2019; Zou, Yuan, et al., 2015), seed‐based functional connectivity (Costumero et al., 2020), and topological properties of the static (Xu et al., 2014) and dynamic (Weng et al., 2020) brain network. These between‐condition differences mainly focus on VN (Costumero et al., 2020; Marx et al., 2004), sensorimotor network (SMN) (Liang et al., 2014), and DMN (Jao et al., 2013), which are situated at the ends of the principal gradients of macroscale resting‐state cortical organization (Margulies et al., 2016). Therefore, EC and EO conditions during the resting state are ideal physiological models to test the efficacy of the gradients approach in within‐subject designs. Differentiating EC and EO can not only test the efficacy of functional gradients in within‐subject conditions but also provide additional information for future resting‐state studies from the prospect of functional hierarchy.

Functional gradients have been used to reveal the ordered changes of specific brain regions along primary axes (Haak et al., 2018; Marquand et al., 2017) and identify biomarkers for clinical samples (Caciagli et al., 2022; Hong et al., 2019). While these studies supported the potential utility of these low‐dimension representations, the reliability of functional gradients in different resting states remains unclear. Therefore, we examined the reliability of functional gradients under EC and EO to provide information for identifying reliable changes and biomarkers while applying functional gradients to resting‐state data.

Here we applied diffusion embedding to differentiate EO and EC in a relatively big sample of 145 participants. We estimated the within‐ and between‐network dispersion of seven well‐established functional brain networks (Yeo et al., 2011) in the context of principal gradients and compared the difference between EC and EO. We further evaluated the test–retest reliability of within‐ and inter‐network dispersion during these two conditions.

## 2 MATERIALS AND METHODS

### 2.1 Participants

We used the public EC/EO datasets in this study (https://www.nitrc.org/projects/eceo_rsfmri_9/). All participants were right‐handed and had no history of neurological or psychiatric disorders. They were recruited after signing written informed consent approved by the local Ethics Committee of research centers. During the resting‐state scans, participants were asked to relax with EC or EO in the scanner with foam pads and strap to minimize the head movement. And the two resting‐state scans (EC and EO) were performed for each subject by counter‐balanced order.

### 2.2 Neuroimaging data acquisition

Neuroimaging data has been described in previous studies (Liu et al., 2013; Yuan et al., 2014, 2018; Zhao et al., 2018; Zou, Yuan, et al., 2015). Details about the neuroimaging data acquisition for all datasets can be found at https://www.nitrc.org/projects/eceo_rsfmri_9/. Dataset1_3, Dataset4, Dataset6, Dataset7, and Dataset8 were used for within‐subject comparisons between EC and EO. And Dataset1_3 and Dataset1_3_V2 were collected with an interval of 8 months and used for the computation of intraclass correlation coefficient (ICC) for the network measures used in this study. Participants in dataset 5 underwent a faster scanning than those in other datasets (1_3, 4, 6, 7, 8). To guarantee the same scanning parameters in our study, we did not include dataset 5 here.

Dataset1_3 and Dataset1_3_V2

Functional magnetic resonance imaging (fMRI) images were acquired using a GE healthcare MR‐750 3T scanner (GE Medical Systems, Milwaukee, WI) with an eight‐channel head coil at Hangzhou Normal University. The images were acquired using a gradient echo Echo‐Planar Imaging (EPI) pulse sequence with the following parameters: TR/TE = 2000/30 ms, flip angle = 60°, slice number = 43 slices, thickness/gap = 3.4/0 mm, FOV = 220 × 220 mm^2^ with an in‐plane resolution of 3.44 × 3.44 mm^2^. The duration of the fMRI scan was 8 minutes, and it included 240 volumes.

Dataset 4

fMRI images were acquired using the same scanner and sequence as Dataset1_3 but with different parameters: TR/TE = 2000/30 ms, flip angle = 60°, slice number = 37 slices, thickness/gap = 3.4/0 mm, FOV = 220 × 220 mm^2^ with an in‐plane resolution of 3.44 × 3.44 mm^2^. The duration of the fMRI scan was 8 min, and it included 240 volumes.

Dataset 6

fMRI images were acquired using a Siemens TRIO 3T scanner at Beijing Normal University. The images were acquired using an EPI sequence with the following parameters: TR/TE = 2000/30 ms, flip angle = 90°, slice number = 33 slices, thickness/gap = 3.5/0.7 mm, FOV = 200 × 200 mm^2^ with an in‐plane resolution of 3.1 × 3.1 mm^2^. The duration of the fMRI scan was 8 min, and it included 240 volumes.

Dataset 7

fMRI images were acquired using the same scanner and sequence as Dataset1_3 but with different parameters: TR/TE = 2000/30 ms, flip angle = 90°, slice number = 43 slices, thickness/gap = 3.2/0 mm, FOV = 220 × 220 mm^2^ with an in‐plane resolution of 3.44 × 3.44 mm^2^. The duration of the fMRI scan was 8 min, and it included 240 volumes.

Dataset 8

fMRI images were acquired using the same scanner and sequence as Dataset1_3 but with different parameters: TR/TE = 2000/30 ms, flip angle = 90°, slice number = 33 slices, thickness/gap = 3.6/0 mm, FOV = 220 × 220 mm^2^ with an in‐plane resolution of 3.125 × 3.125 mm^2^. The duration of the fMRI scan was 8 min, and it included 240 volumes.

### 2.3 Neuroimaging preprocessing

We preprocessed resting‐state fMRI using the Statistical Parametric Mapping 12 (SPM12) (https://www.fil.ion.ucl.ac.uk/spm/software/spm12/). Preprocessing steps included realignment for head motion correction, slice timing, coregistering to the high‐resolution three‐dimensional T1‐weighted images, segmentation of six tissue possibility templates from SPM12, and normalization via T1 images (resampled to 3 × 3 × 3 mm^3^). Then we smoothed the normalized functional images with an 8‐mm full width at half maximum Gaussian kernel. We further regressed out the time series of white matter (WM, 99% probability SPM map) as well as cerebrospinal fluid (CSF, 90% probability SPM map) (Zang et al., 2018), global mean time course, six head motion parameters from the realignment step, and the frame‐wise displacement (FD) (Power et al., 2012). Lastly, we applied a band‐pass filtering with 0.01–0.1 Hz.

### 2.4 Construction of functional gradients and network dispersion

The original description of functional gradients was at vertex‐wise level, implying decomposing a matrix with a dimension of tens of thousands. To gain high spatial resolution while reducing the computational burden, we randomly segregated the entire cerebral cortex into 3378 brain areas using the compact parcellation approach (Zalesky et al., 2010). The parcellated 3378 regions were spatially randomly distributed and the largest node (540 mm^3^) was no larger than twice of the smallest node (270 mm^3^). We used Pearson's correlation coefficient to construct individual functional connectivity matrix and applied zscore normalization to mitigate the differences caused by multicenter datasets. We built the group‐template connectivity matrix by averaging the connectivity matrix of all scans during both EC and EO, Fisher *z*‐transformed it and thresholded it by remaining only the top 10% connection of each column (Margulies et al., 2016).

We built the affinity matrix using cosine similarity and then applied diffusion embedding to it to obtain the gradients template with BrainSpace toolbox (Vos de Wael et al., 2020). Then we aligned individual gradients to the group template using Procrustes analysis and calculated the dispersion of seven brain networks as before (Bethlehem et al., 2020). Within‐network dispersion was defined by computing the Euclidean distance between all nodes within one network and the centroid of this network, while between‐network dispersion was defined by computing the distance between the centroid of one network and the centroids of all other networks.

### 2.5 Statistical analysis

We applied the repeated measures analysis of variance (ANOVA) to compare the within‐subject differences in FD, within‐ and between‐network dispersion of seven networks between EC and EO using Statistical Product and Service Solutions 26 (https://www.ibm.com/analytics/spss-statistics-software). Furthermore, to explore whether the differences in between‐network dispersion result from certain networks or all other networks, we computed the dispersion between one network and other six networks separately and then compared them between EC and EO one by one. After that, we corrected for multiple comparisons using Bonferroni correction.

To evaluate the reliability of the network gradients and network dispersions, we computed the ANOVA ICCs for the network gradients and within‐ and between‐network dispersions of seven networks according to the following equation (Shrout & Fleiss, 1979):

$$\mathrm{ICC}=\left(\mathrm{BMS}-\mathrm{EMS}\right)/\left(\mathrm{BMS}+\left(K-1\right)*\mathrm{EMS}\right)$$

where BMS represents between‐subject mean square, EMS represents the error mean square, and *K* is the number of repeated sessions.

Negative ICCs implying negative reliability are theoretically difficult to interpret (Rousson et al., 2002) and the reason for negative ICCs is unclear (Müller & Büttner, 1994). Some researchers suggested that negative ICCs are consequences of bad estimates and that increasing the sample size may avoid them (Liljequist et al., 2019). To avoid negative ICCs, we also utilized linear mixed models to assess both the intra‐ and interparticipant variability and estimated the model variances with the restricted maximum likelihood (ReML) approach while computing ICCs (Zuo et al., 2013). The ReML ICCs were calculated via DPABI (Yan et al., 2016). Statistical difference between EC and EO was assessed with repeated‐measure ANOVA using eyes condition as the factor.

Lastly, we validated the main analysis by utilizing a different spatial‐scale brain parcellation‐Brainnetome atlas (Fan et al., 2016) and using a different gradients alignment method‐joint embedding (Vos de Wael et al., 2020).

Codes that are used to compute the functional gradients can be found at GitHub (https://github.com/XlZha/Gradients_HBM).

## 3 RESULTS

### 3.1 Sample characterization

We found significant within‐subject difference in FD (*F*(1,144) = 17.873, *p* = 4.2 × 10^−5^) between EC and EO. To exclude the effects of FD on the results, we regressed out the FD during preprocessing neuroimaging data and controlled for the difference between FD during EC and EO in the following analyses. See Table 1 for demographic characteristics.

**TABLE 1:** Demographic characteristics for all datasets

	*n*	Age, mean (*SD*)	Sex, F/M	Handedness	FD, mean (*SD*)	
					EC	EO
dataset1_3	21	21.67 (1.71)	11/10	Right	0.053 (0.024)	0.044 (0.014)
dataset4	33	23.48 (2.05)	18/15	Right	0.054 (0.027)	0.046 (0.016)
dataset6	43	22.23 (1.64)	22/21	Right	0.065 (0.027)	0.06 (0.022)
dataset7	28	23.43 (2.73)	15/13	Right	0.043 (0.018)	0.04 (0.016)
dataset8	20	20.95 (1.82)	10/10	Right	0.06 (0.025)	0.058 (0.029)

Abbreviations: EC, eyes closed; EO, eyes open; F, female; FD, framewise displacement; M, male; *n*, number; *SD*, standard deviation.

### 3.2 Network dispersion comparisons

**FIGURE 1:**
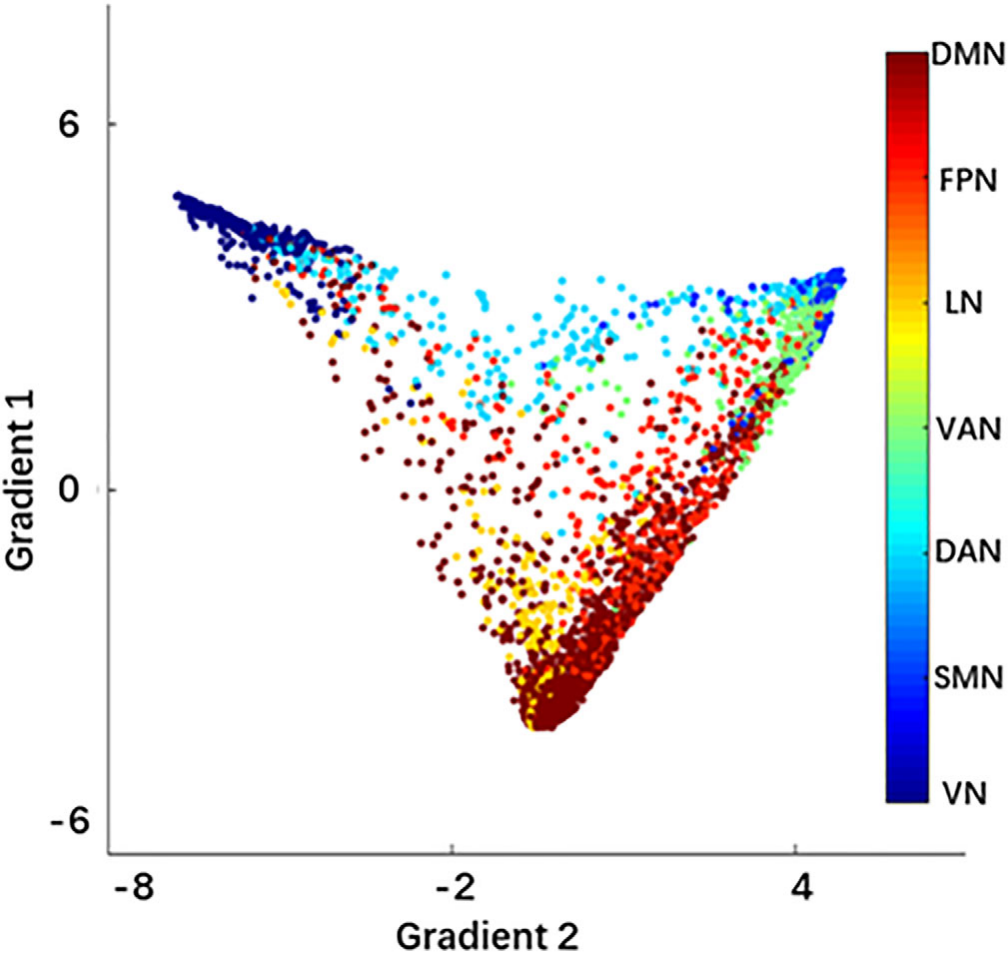
Group template of the first two gradients. The first gradient ran from unimodal to transmodal cortex, while the second gradient ran from VN to SMN. DAN, dorsal attention network; DMN, default mode network; FPN, frontoparietal network; LN, limbic network; SMN, sensorimotor network; VAN, ventral attention network; VN, visual network

By applying diffusion embedding to the group‐averaged connectivity matrix, we obtained a group template of the principal gradients of the macroscale cortical organization. The first gradient explained 13.53% of variance and ran from unimodal to transmodal cortex, while the second gradient explained 12.98% of variance and ran from VN to SMN (Figure 1), which is consistent with the original description of the principal gradients (Margulies et al., 2016).

Before comparing network dispersion between EC and EO, we first compared the within‐subject difference in the explanation ratios of the principal gradients between eyes conditions. Neither the first (*F*(1,144) = 1.002, *p* = .318) nor the second (*F*(1,144) = 3.606, *p* = .06) gradient showed significant within‐subject difference between EC and EO, implying that the following within‐subject comparisons would not be affected by explanation ratios.

**FIGURE 2:**
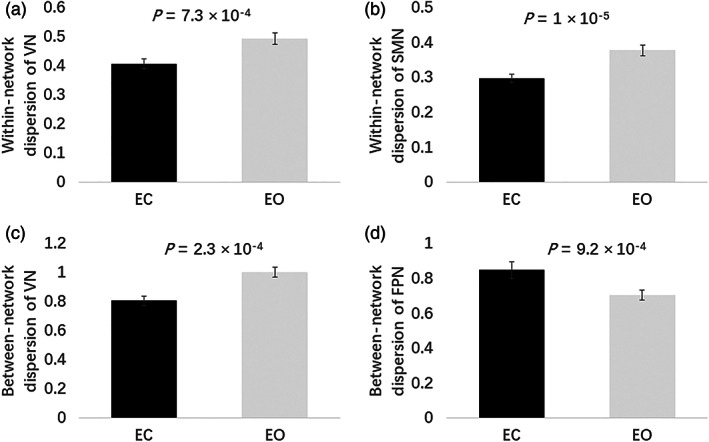
Within‐subject differences in within‐ and between‐network dispersion between eyes closed (EC) and eyes open (EO). (a) Visual network (VN) and (b) sensorimotor network (SMN) showed significantly lower within‐network dispersion during EC than EO. (c) VN showed significantly lower and (d) frontoparietal network (FPN) showed significantly higher between‐network dispersion under EC than EO

**FIGURE 3:**
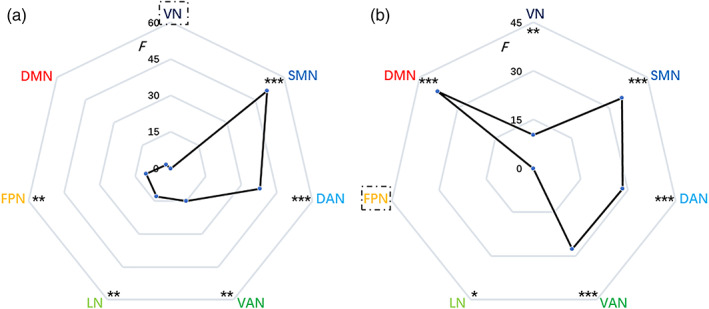
Between‐condition differences in between‐network dispersion between VN (a) as well as FPN (b) and other networks. DAN, dorsal attention network; DMN, default mode network; F, *F* value for repeated measures analysis of variance; FPN, frontoparietal network; LN, limbic network; SMN, sensorimotor network; VAN, dorsal attention network; VN, visual network. *uncorrected *p* < .05; **p_Bonferroni_ < .05; ***p_Bonferroni_ < .001

Then we used repeated measures ANOVA to compare the within‐ and between‐network dispersion of the seven networks. VN (*F*(1,143) =11.933, *p* = 7.3 × 10^−4^ < *p*_Bonferroni_ = .0036, Figure 2a) and SMN (*F*(1,143) = 20.947, *p* = 1 × 10^−5^ < *p*_Bonferroni_ = .0036, Figure 2b) both showed significantly lower within‐network dispersion during EC than EO after correcting for FD and multiple comparisons. And VN showed significantly lower between‐network dispersion under EC than EO (*F*(1,143) = 14.324, *p* = 2.3 × 10^−4^ < *p*_Bonferroni_ = .0036, Figure 2c), while frontoparietal network (FPN) showed significantly higher between‐network dispersion under EC than EO (*F*(1,143) = 11.447, *p* = 9.2 × 10^−4^ < *p*_Bonferroni_ = .0036, Figure 2d) after correcting for multiple comparisons. What is more, we found the between‐condition differences in between‐network dispersion were general to other networks (Figure 3).

We replicated the main analysis by applying joint embedding to align individual gradients to the group template: VN (*F*(1,143) = 8.933, *p* = .0033 < *p*_Bonferroni_ = .0036) and SMN (*F*(1,143) = 22.681, *p* = 5 × 10^−6^ < *p*_Bonferroni_ = .0036) both showed significantly lower within‐network dispersion during EC than EO; VN showed significantly lower between‐network dispersion under EC than EO (*F*(1,143) = 16.352, *p* = 8.6 × 10^−5^ < *p*_Bonferroni_ = .0036), while FPN showed significantly higher between‐network dispersion under EC than EO (*F*(1,143) = 16.787, *p* = 7 × 10^−5^ < *p*_Bonferroni_ = .0036). Therefore, the alignment methods did not affect our main results.

While using the Brainnetome atlas, we also found SMN (*F*(1,143) = 7.41, *p* = .007) showed significantly lower within‐network dispersion and FPN (*F*(1,143) = 6.921, *p* = .009) showed significantly higher between‐network dispersion during EC than EO. The within‐ (*F*(1,143) = 0.906, *p* = .343) and between‐network (*F*(1,143) = 2.358, *p* = .127) dispersion of VN did not show any significant difference. Other significant within‐subject differences include higher within‐network dispersion of dorsal attention network (*F*(1,143) = 12.234, *p* = .001) and ventral attention network (*F*(1,143) = 11.586, *p* = .001), lower within‐network dispersion of FPN (*F*(1,143) = 3.943, *p* = .049); lower between‐network dispersion of limbic network (*F*(1,143) = 6.425, *p* = .012) and DMN (*F*(1,143) = 9.732, *p* = .002) during EC than EO. The reason why only part of the results was replicated may be that the functional network with 3378 nodes better captures the functional hierarchy of the original vertex‐wise connectivity matrix, in consideration of smoothing the signals of voxels during preprocessing neuroimaging data and more nodes within the connectivity matrix.

### 3.3 Reliability of network dispersion

Lastly, we computed the ICCs for network gradients and the within‐ and between‐network dispersions across the seven networks under EC and EO (Table 2). The network dispersions showed relatively low reliability when compared to network gradients. Only the within‐network dispersion of the SMN under EO showed moderate reliability (ICC = 0.407), while all other network dispersion under EC or EO showed fair reliability (ICC <0.4). Using repeated‐measure ANOVA, we found that ICCs for the within‐network dispersion (*F*(1,6) = 2.25, *p* = .184) under EO were higher but not significantly higher than those under EC, while the ICCs for between‐network dispersion (*F*(1,6) = 0.34, *p* = .581) were almost the same between EC and EO. We also listed the ReML ICCs in Table 2, which showed similar values except that negative ANOVA ICCs were replaced with very small positive numbers.

**TABLE 2:** ICCs for network gradients and dispersions across seven networks under EC and EO

		VN	SMN	DAN	VAN	LN	FPN	DMN	Mean (*SD*)
ANOVA ICC									
Network gradients	EC_G1	0.135	0.391	0.172	0.442	0.105	0.314	0.34	0.271 (0.13)
	EC_G2	0.306	−0.094	0.494	0.266	0.35	0.032	0.226	0.226 (0.2)
	EO_G1	0.022	0.373	0.304	0.274	0.408	0.235	0.311	0.275 (0.13)
	EO_G2	0.299	0.072	0.648	−0.155	0.46	0.169	0.165	0.237 (0.26)
Network dispersion	EC_within	0.225	0.06	0.099	−0.13	−0.047	0.199	−0.016	0.056 (0.13)
	EC_between	−0.135	0.207	−0.102	0.206	0.092	0.07	0.247	0.084 (0.15)
	EO_within	−0.152	0.407	0.231	0.262	0.139	0.258	0.276	0.203 (0.18)
	EO_between	0.121	0.103	0.145	0.035	0.204	0.01	0.231	0.121 (0.08)
ReML ICC									
Network gradients	EC_G1	0.142	0.386	0.177	0.424	0.101	0.336	0.338	0.272 (0.13)
	EC_G2	0.232	0	0.509	0.288	0.36	0.034	0.242	0.238 (0.18)
	EO_G1	0.046	0.393	0.321	0.284	0.384	0.253	0.325	0.287 (0.12)
	EO_G2	0.32	0.086	0.657	0	0.446	0.181	0.184	0.268 (0.23)
Network dispersion	EC_within	0.248	0.084	0.117	0	0	0.222	0	0.096 (0.11)
	EC_between	0	0.217	0	0.195	0.103	0.088	0.255	0.123 (0.1)
	EO_within	0	0.42	0.243	0.282	0.161	0.247	0.255	0.23 (0.13)
	EO_between	0.144	0.127	0.167	0.049	0.22	0.031	0.241	0.14 (0.08)

*Note*: Here the 0 in table means very small positive number close to 0.

Abbreviations: ANOVA, analysis of variance; DAN, dorsal attention network; DMN, default mode network; EC, eyes closed; EO, eyes open; FPN, frontoparietal network; G1, gradient 1; G2, gradient 2; ICC, intraclass correlation coefficient; LN, limbic network; ReML, restricted maximum likelihood; *SD*, standard deviation; SMN, sensorimotor network; VAN, ventral attention network; VN, visual network.

## 4 DISCUSSION

In this study, we mainly tested whether diffusion embedding could differentiate two resting‐state within‐subject conditions: EC and EO. Diffusion embedding was applied to the resting‐state functional connectivity matrix to obtain the principal gradients of cortical network organization. Then we used within‐network dispersion to depict the heterogeneity of connectivity pattern within one functional network and between‐network dispersion to describe the difference in connectivity pattern between functional networks. We found lower within‐network dispersion of VN and SMN under EC than EO. We also discovered lower between‐network dispersion of VN and higher between‐network dispersion of FPN under EC than EO. Lastly, we computed ICC to measure the reliability of network dispersion used here. Most of the within‐ and between‐network dispersion of seven networks exhibited fair reliability and the reliability under EO was higher than reliability under EC.

### 4.1 Network dispersion comparisons between EC and EO

First, we found significantly lower within‐ and between‐network dispersion of VN under EC than EO. Lower within‐network dispersion means functional connectivity pattern of VN is more similar under EC than EO, while lower between‐network dispersion means VN is more incorporated into the global functional connectivity under EC than EO. The changed network dispersion of VN from EO to EC might result from visual imagery during relaxation (Bianciardi et al., 2009). And previous study revealed increased connections between VN and motor, somatosensory as well as auditory networks under EC compared to EO, while the connections between VN and attention as well as arousal networks were decreased under EC compared to EO (Costumero et al., 2020; Xu et al., 2014). The decreased within‐ and between‐network dispersion of VN suggests a gradient compression pattern of the visual system in the cortical hierarchy organization and that the visual system plays a more homogeneous and central role‐visual imagery during EC (Bianciardi et al., 2009), from the perspective of the cerebral hierarchical organization rather than visual system along.

We also found significantly lower within‐network dispersion of SMN during EC than EO, which might originate from mental imagery (Mazard et al., 2002, 2005) or EC allows more time to improve perception and focus on sensation, leading to increased intra‐ and inter‐network connectivity of SMN (Agcaoglu et al., 2019). Multidimensional evidences indicated that spontaneous brain activity is effectively associated with EC and EO resting states, and sensorimotor and occipital regions show opposite brain activity (Wei et al., 2018). Similarly, the lower within‐network dispersion of SMN suggests a gradient compression pattern of the sensorimotor system in the cortical hierarchy organization, which may serve the function of mental imagery more homogeneously during EC (Mazard et al., 2005).

In a word, the gradient compression patterns of visual and sensorimotor systems imply a suppression of sensory function during EO (Xu et al., 2014) and suggest that EC can be regarded as an “interoceptive” mental state characterized by imagery and multisensory function (Marx et al., 2003).

Apart from VN and SMN, we also discovered significantly higher between‐network dispersion of FPN during EC than EO, implying that FPN is closer to the center of global connectivity axis during EO. From a more integrative perspective of the cortical hierarchy organization, the increased between‐network dispersion of FPN from EO to EC may emphasize the important role of this cognitive system during EO ‐ processing the exteroceptive information (Hüfner et al., 2009).

Altogether, these results support that EO corresponds to a suppression of sensory function to allocate resources to exteroceptive processing, while EC leads to an “interoceptive” mental state characterized by multisensory activity (Hüfner et al., 2009; Marx et al., 2004; Xu et al., 2014). Functional gradient provides a more integrated vision of the brain activity changes between EC and EO by capturing the continuous spatial patterns of functional connectivity beyond segregated brain systems and also provides a simplified description of the principal dimensions to characterize the alteration of the macroscale cortical hierarchy from EC to EO. In addition, future studies should pay attention to the state of their data (EC or EO) and be careful while explaining their findings.

### 4.2 Reliability of network dispersion

Second, we identified fair ICC for most of the within‐ and between‐network dispersion of seven networks, suggesting that the measures we used to differentiate within‐subject EC and EO conditions are not that reliable. Previous intrasession study has reported that functional connectivity of different networks showed moderate‐to‐high reliability during EC and EO (Patriat et al., 2013). And network‐wise gradients also showed moderate‐to‐high reliability (Hong et al., 2020). These suggest that the reliability of network dispersion was relatively low. What is more, we found higher but not significantly higher reliability under EO than EC, which is consistent with previous findings that EO showed higher test–retest reliability and greater stability (Weng et al., 2020; Zou, Miao, et al., 2015). Some studies set negative ANOVA ICCs to zero (Braun et al., 2012; Zhang et al., 2011), but using ReML approach while estimating the model variances can help avoid negative ICC values.

### 4.3 Limitations

This study has several limitations. First, the sample size we used for ICC computation was small. Therefore, further studies with bigger sample size are needed to validate the findings (Chen et al., 2018) and may avoid negative ICCs (Liljequist et al., 2019). Second, the participants were all young, which limit the generality of our results. Considering the development of principal functional gradients across lifespan (Bethlehem et al., 2020; Dong et al., 2021; Nenning et al., 2020), it is necessary to replicate the analyses using datasets with different age ranges. Thirdly, we only included cortical regions in the study because of the original description of the principal gradients of cortical network organization (Margulies et al., 2016). Considering the significantly changed functional connectivity of thalamus (Zou et al., 2009), further study should also include subcortical regions like thalamus.

## 5 CONCLUSIONS

Here, we evaluated both the dynamic differences and the reliability of EC and EO to try to fill the gap in the current studies on functional gradients. We not only confirmed the efficacy of functional gradients in within‐subject conditions, but also suggested increasing the sample size to gain reliable biomarkers in those within‐subject studies.

In sum, the results showed that even the reliability of gradients‐based measures used here was relatively low, within‐ and between‐network dispersion can differentiate within‐subject conditions and help find biologically meaningful differences between two common resting‐state conditions: EC and EO that align well with previous findings. Diffusion embedding can not only be applied to between‐subject conditions, such as brain diseases and neurodevelopment, but also be generalized to within‐subject conditions. Our study expands the application of diffusion embedding.

## AUTHOR CONTRIBUTIONS

Xiaolong Zhang and Zhenxiang Zang conceived the experiments, Xiaolong Zhang analyzed the results and wrote the manuscript, Xiaolong Zhang and Zhenxiang Zang reviewed the manuscript.

## CONFLICT OF INTEREST

The authors reported no biomedical financial interests or potential conflicts of interest.

## Data Availability

Link to the data can be found in Section 2. Codes can be found at GitHub (https://github.com/XlZha/Gradients_HBM).
